# Fusion of glioma-associated mesenchymal stem/stromal cells with glioma cells promotes macrophage recruitment and M2 polarization via m^6^A modification of CSF1

**DOI:** 10.1038/s41419-025-07678-x

**Published:** 2025-04-26

**Authors:** Zhen Liu, Sujie Gu, Zesheng Peng, Yihao Wang, Hui Li, Xiaoqing Zeng, Haofei Wang, Peng Lv, Yuyi Wu, Yan Zhou, Yanbin Zhang, Xiaobing Jiang, Peng Fu

**Affiliations:** 1 Department of Neurosurgery, Union Hospital, Tongji Medical College, Huazhong University of Science and Technology, Wuhan, 430022 China; 2 Department of Neurosurgery, Henan Provincial People’s Hospital, Zhengzhou, 450000 China; 3 Department of Cataract, Nanyang Eye Hospital, Nanyang, 473000 China; 4 Department of Infectious Diseases, Union Hospital, Tongji Medical College, Huazhong University of Science and Technology, Wuhan, 430022 China

**Keywords:** CNS cancer, Cancer microenvironment

## Abstract

Malignant glioma is the most common primary malignant tumor of the brain in adults, with glioblastoma (GBM) being the most aggressive subtype. Mesenchymal stem/stromal cells (MSCs) have been shown to fuse with tumor cells in various cancers including glioma, thereby regulating tumor progression. However, there has been no systematic research on the fusion of glioma-associated MSCs (GA-MSCs) with glioma cells. Here, it is shown that GA-MSCs are able to spontaneously fuse with glioma cells both in vitro and in vivo. The hybrid cells display significantly lower levels of N6-methyladenosine (m^6^A) modification and can modulate the glioma microenvironment by attracting and inducing M2-like polarization of macrophages. Mechanistically, the demethylase fat mass and obesity-associated protein (FTO) mediates demethylation in hybrids and promotes macrophage colony-stimulating factor (CSF1) secretion by increasing its RNA stability in an m^6^A-YTH domain family 2 (YTHDF2)-dependent manner. Our study reveals a novel crosstalk mechanism between glioma cells, GA-MSCs, and macrophages in glioma microenvironment, offering potential new approaches for glioma therapy.

## Introduction

Glioma is the most prevalent primary intracranial malignancies among adults, and are characterized by high proliferation rates and notably poor prognoses [1]. Glioblastoma (GBM), the most aggressive glioma subtype, accounts for roughly half of all malignant brain tumors. Despite multimodal therapeutic approaches, including microsurgery, radiotherapy, temozolomide-based chemotherapy, and tumor-treating fields, the median overall survival of GBM patients remains less than 2 years [2]. The initiation and maintenance of gliomas result from intricate interactions involving multiple factors, with a particular emphasis on the interplay between gliomas and their tumor microenvironment (TME) [3]. The glioma TME includes stromal cells, immune cells, signaling molecules, and the extracellular matrix. The complex and dynamic interplay between glioma cells and diverse cellular components within the TME fosters tumor invasiveness, angiogenesis, therapeutic resistance, and recurrence [4].

Mesenchymal stem/stromal cells (MSCs) are multipotent cells that were originally identified in the bone marrow. Subsequent investigations have demonstrated their presence in the perivascular microenvironment of virtually all organs and tissues, including tumor tissues [5, 6]. Compared with MSCs in normal tissues, MSCs in the TME undergo tumor education, resulting in distinct characteristics [7]. MSC-like cells have also been found present in glioma, referred to as glioma associated MSCs (GA-MSCs). Further research has shown that GA-MSCs promote glioma progression and are strongly associated with poor prognosis [8, 9]. Tumor-associated MSCs contribute to TME formation through multiple mechanisms, such as immune suppression, angiogenesis promotion, and differentiation into diverse cell lineages [7]. Additionally, MSCs can directly interact with surrounding cells via various means, including paracrine cytokine secretion, vesicular exchange, and cell fusion [10].

Cell-cell fusion is an essential process in physiological development and has been recognized to contribute to tumorigenesis and tumor progression for more than a century [11, 12]. Through cell fusion, tumor cells undergo rapid nuclear reprogramming and epigenetic modifications, significantly enhancing tumor heterogeneity and progression [13, 14]. The fusion of cancer cells and MSCs modulates tumor maintenance and evolution in diverse malignancies, including breast cancer, gastric cancer, ovarian cancer, and liver cancer [15]. Glioma cells have also been observed to fuse with bone marrow-derived MSCs (BMSCs), leading to increased tumor proliferation, neovascularization, and invasion propensity [16–18].

Previous studies have shown that cell fusion leads to nuclear reprogramming, including epigenetic changes like DNA methylation [19, 20]. Epigenetics modifications, such as DNA and RNA methylation, histone modification and noncoding RNA regulation, have profound impacts on heredity, growth and diseases. As the most prevalent RNA modification in humans, N6-methyladenosine (m^6^A) plays a pivotal role in both physiological and pathological conditions, particularly in cancer initiation and progression [21]. However, alteration in m^6^A modification following the fusion of tumor cells with MSCs remain poorly understood.

Here, we aimed to investigate the changes in the m^6^A modification of GBM cells after fusion with MSCs, and the resulting effects on tumor progression. Furthermore, in our previous studies, we successfully isolated and identified GA-MSCs from surgical specimens [22, 23]. Compared to BMSCs, GA-MSCs are more suitable candidates for exploring the interaction between MSCs and tumors, as they directly contact tumor cells and have already been educated by tumor.

In this study, our findings confirmed that glioma cells can fuse with GA-MSCs. Compared with the parental GBM cells, newly formed cells resulting from the fusion of GBM cells and GA-MSCs, termed hybrids, exhibit lower levels of m^6^A modification. Subsequent results suggested that this reduction in m^6^A levels was mediated by fat mass and obesity-associated protein (FTO). Hybrids showed an enhanced ability to stimulate macrophage recruitment and M2 polarization, thus promoting GBM growth in vivo. Further data demonstrated that reduced m^6^A levels stabilize macrophage colony-stimulating factor (CSF1) mRNA, which is recognized by YTH domain family 2 (YTHDF2). The elevated levels of CSF1 derived from hybrid cells reprogram the glioma TME by modulating macrophages, consequently promoting glioma progression.

## Materials and methods

### Bioinformatics analysis

Single cell RNA-seq (scRNA-seq) data was downloaded from the Gene Expression Omnibus (GEO) database (GSE131928). Data from three patients (MGH124, MGH125 and MGH143) were selected for further analysis. The data were analyzed in R software (version 4.2.3) and converted to Seurat objects using the Seurat package (version 4.3.0) [24]. Cells were filtered based on mitochondrial gene content (>10%) and the number of detected genes (>8000 or <200). The SingleR (2.0.0) and copykat (version 1.1) packages were used for cell annotation [25, 26]. A total of 6337 cells were obtained, with 397 cells identified as stroma-like cells (including MSCs) based on CXCL2 and S100A4 expression.

### Human GBM specimens

Human GBM specimens were collected from patients who were pathologically diagnosed with GBM at Wuhan Union Hospital. All the patients involved provided informed consent. The clinical information of the GBM patients is listed in Supplementary Table S1. This study was approved by the Institutional Ethics Committee of Tongji Medical College, Huazhong University of Science and Technology.

### Isolation of GA-MSCs, cell culture and transfection

The isolation and identification of GA-MSCs were conducted as described in our previous study, and the patient information involved in this study is presented in Supplementary Table S1 [22, 23]. U87MG (CL0238), U251 (CL0237), THP-1 (CL0223), and RAW264.7(CL0190) cells were purchased from Procell Life Science & Technology (China). Cells were used within six months of culturing and tested every two months to ensure they were free of Mycoplasma contamination. THP-1 cells were cultured in specialized media (CM0223, Procell Life Science & Technology, China), and other cells were cultured in Dulbecco’s modified Eagle’s medium (DMEM, Cytiva, USA) with 10% fetal bovine serum (Biological Industries, Israel) and 1% penicillin-streptomycin Solution (Biosharp, China) at 37 °C in a 5% CO2 atmosphere. THP-1-macrophages were derived from THP-1 cells by treatment with 100 ng/ml of phorbol 12-myristate 13 acetate (PMA, MedChemExpress, China) for 48 h.

Green fluorescent protein (GFP) expressing GBM cells with hygromycin resistance and GA-MSCs-mCherry with blasticidin resistance were generated via lentivirus transfection (GeneChem, China). FTO and YTHDF2 knockdown lentiviruses (with GFP and puromycin resistance genes) and their negative controls were also purchased from GeneChem. In brief, lentivirus (MOI = 5) and 1X HitransG P (GeneChem) were added to the cell culture medium. After 16 h of incubation, the medium was replaced with fresh culture medium. The cells were then cultured for an additional 48 h. Subsequently, puromycin (5 μg/ml, Sigma-Aldrich), hygromycin B (500 μg/ml, MedChemExpress, China) or blasticidin (5 μg/ml, Beyotime Biotechnology, China) was added for selection. The cells were then treated with these reagents for one week to establish stable expression/knockdown cells. The target sequences used for the lentiviruses were as follows: shFTO-1: TCACGAATTGCCCGAACATTA, shFTO-2: CGGTTCACAACCTCGGTTTAG, shYTHDE2-1: GCTACTCTGAGGACGATATTC, shYTHDF2-2: AGGCTTTGGTTCAGAATAT. The scrambled shRNA sequence TTCTCCGAACGTGTCACGT was used as a negative control.

### Co-culture experiments

GBM cells expressing GFP and resistance to hygromycin were mixed with GA-MSCs expressing mCherry and resistance to blasticidin at a 4:1 ratio and co-cultured. In the in vitro experiments, the cells were directly observed under a fluorescence microscope (Olympus IX71, Japan) after 24 h of co-culture. Hybrids were selected by adding 500 μg/ml hygromycin B and 5 μg/ml blasticidin to the co-culture medium.

For in vivo experiments, the mixed cell population (totaling 5 × 10^5^ cells) was injected into the brains of mice immediately after mixing (refer to the “Intracranial xenograft model” section for detailed procedures). Three weeks later, the mice were euthanized and their brain tissues were collected. The tissues were embedded in optimal cutting temperature compound embedding agent (Servicebio, China), sectioned at 8 μm thickness, and observed under a fluorescence microscope.

### 5-Ethynyl-2′-deoxyuridine (EdU)-labeling assay

Glioma cells were incubated with 10 μM EdU for 24 h and their nuclei were labeled with Alexa Fluor 488 according to the protocol of the BeyoClick™ EdU Cell Proliferation Kit (Beyotime Biotechnology, China). GA-MSCs were treated with 1 μM Celltracker CM-DiI (Yeasen Biotechnology, China) for 10 min, and then washed three times with PBS to ensure the complete removal of any residual dye. The two cell types were then co-cultured for 12 h. Subsequently, the nuclei were stained with Hoechst 33342 for 10 min and observed under a fluorescence microscope.

### Western blot

The protein concentration was determined using a BCA protein assay kit (Beyotime Biotechnology, China) according to the manufacturer’s protocol. Proteins were separated on 4–12% SDS-PAGE gels and transferred to PVDF membranes (Millipore, USA). The membranes were blocked with 5% nonfat milk at room temperature for 1 h and then incubated with primary antibodies at 4 °C overnight. After three washes with TBST, the membranes were treated with horseradish peroxidase-conjugated secondary antibodies for 2 h. Finally, enhanced chemiluminescence (ECL) reagent (G2161, Servicebio, China) was used to visualize protein expression using the chemiluminescent imaging system (OI600 Touch, Guangyi Biotechnology, China). All antibodies used in the experiments are listed in Supplementary Table S2. All original images of the Western blots are provided in Additional file 3.

### RNA extraction and quantitative real-time polymerase chain reaction (qPCR)

Total RNA from each cell sample was extracted using TRIzol reagent (Invitrogen), and cDNA was synthesized by reverse transcription following the manufacturer’s instructions using a reverse transcription kit (RR036A, Takara). qPCR was conducted on a LightCycler 480 Real-Time PCR system using TB Green® Premix Ex Taq™ II (RR820A, Takara). GAPDH served as the normalization control, and the comparative Ct method (ΔΔCt) was used to evaluate mRNA expression. The primers used in this study are listed in Supplementary Table S3.

### RNA m^6^A dot blot assay and quantification

Total RNA was isolated from various cells as previously described. The m^6^A dot blot assay was conducted according to a published protocol [27]. Briefly, total RNA from all groups was quantitatively diluted to the same concentration and heated at 95 °C for 3 min. Then the samples were loaded onto a nylon membrane (INYC00010, Millipore) and crosslinked using an ultraviolet crosslinker. The membrane was then blocked and incubated with an anti- m^6^A antibody (1:1000 dilution, 91261, Active Motif, USA) overnight at 4 °C. Afterward, the membrane was incubated with a secondary antibody at room temperature, and detected using ECL detection reagent (G2161, Servicebio). Methylene blue staining was used as a control to ensure equal total RNA concentrations.

For quantification of RNA m^6^A levels, we used a m^6^A RNA methylation quantification kit (p-9005, EpigenTek, USA) following the manufacturer’s instructions. The m^6^A content was quantified by measuring the absorbance at 450 nm.

### Cell proliferation

For the CCK-8 assay (MedChemExpress, China), cells were plated at a density of 1000 cells per well in 96-well plates to evaluate cell viability. The plates were incubated at 37 °C for 2 h, after which the absorbance was read at 450 nm.

We also utilized the BeyoClick™ EdU Cell Proliferation Kit (Beyotime Biotechnology, China) to evaluate cell proliferation. After adhering to a 96-well plate, cells were incubated with 10 µM EdU for 4 h. Subsequently, the cells were fixed, permeabilized, and stained with an Alexa Fluor 488 reaction cocktail for EdU and Hoechst 33342 for cell nuclei. Finally, the samples were imaged under a fluorescence microscope.

### Intracranial xenograft model

All animal experiments were approved by the Institutional Animal Care and Use Committee of Huazhong University of Science and Technology and were conducted in accordance with NIH animal care guidelines. Six-week-old male BALB/c-nude mice (BIONT, Wuhan, China) were randomly assigned to experimental groups and used to construct the intracranial xenograft model. Mice that died unexpectedly during the experiment were excluded from the analysis. Briefly, a total of 5 × 10^5^ U87MG or Hybrids cells stably expressing firefly luciferase with the indicated treatments were injected into the mouse brain at 1.5 mm lateral, 2 mm posterior to the bregma, and 2.5 mm in depth using a Hamilton syringe (Hamilton Company, USA). Tumor growth was monitored using bioluminescence imaging (Bruker Corporation, USA). Tumor tissues harvested from the mice were subsequently used for corresponding staining procedures.

For the macrophage depletion experiment, we followed a previously published method [28]. Hybrid86 glioma model mice were randomly divided into two groups, and treated with either liposome-PBS or liposome-clodronate (Yeasen Biotechnology, China). On day 4, the animals received an intraperitoneal injection of 50 mg/kg body weight of liposome-clodronate or an equivalent volume liposome-PBS. Subsequently, the treatment was continued on days 7, 10, 13, 20, and 27 at a reduced dose of 25 mg/kg to mitigate adverse effects. Tumor growth was also monitored using bioluminescence imaging as described previously.

### m^6^A-methylated RNA immunoprecipitation sequencing (m^6^A-seq)

Total RNA from U87MG and Hybrid86 cells was extracted using TRIzol reagent as previously described. Both mRNA-seq and m^6^A -seq were then performed simultaneously (GeneChem, China). For RNA-seq, 1 µg of RNA per sample was used as input material for RNA sample preparation. Sequencing libraries were generated using the NEBNext® UltraTM RNA Library Prep Kit for Illumina® (NEB, USA) according to the manufacturer’s instructions. Subsequently, 2 × 150 bp paired-end (PE150) sequencing was conducted on an Illumina NovaSeq 6000. For m^6^A -seq, the cleaved RNA fragments were incubated with a m^6^A antibody (Synaptic Systems, Germany) in immunoprecipitation buffer. Next, 2 × 150 bp paired-end sequencing was performed on an Illumina NovaSeq 6000, and the results were visualized with IGV software (version 2.16.1) [29].

### Immunofluorescence (IF) and immunohistochemistry (IHC)

For IF, after tissues were fixed with 4% paraformaldehyde, they were paraffin-embedded and sectioned. The sections were then blocked with 3% bovine serum albumin for 30 min, followed by overnight incubation with the primary antibody. After washing, the sections were incubated with the secondary antibody for 50 min. Subsequently, tyramide signal amplification technology was used to fluorece the secondary antibody, and the same protocol was repeated for the next primary and secondary antibodies. Finally, the nuclei were stained with DAPI for 10 min before they were mounted on. The following fluorescent dyes were used for tyramide signal amplification: iF488-tyramide (G1231, Servicebio), iF546-tyramide (G1251, Servicebio), iF555-tyramide (G1233, Servicebio) and iF594-tyramide (G1242, Servicebio).

We employed IHC to determine the localization and expression levels of the proteins. In brief, tissue sections were deparaffinized, hydrated, and subject to antigen retrieval using sodium citrate. Endogenous peroxidase activity was blocked by treating the sections with 3% H_2_O_2_ for 10 min. Subsequently, the sections were incubated with primary antibodies overnight, followed by incubation with secondary antibodies. The signals were visualized using diaminobenzidine (DAB) staining, and the sections were counterstained with hematoxylin. Images from IF and IHC were captured using a fluorescence microscope. DAB staining was semi-quantified using ImageJ Fiji software by determining the percentage of the area that was positive. The percent area fraction was calculated as the percentage of pixels in the image that met the applied thresholds.

### Enzyme linked immunosorbent assay (ELISA)

Equal number of cells were seeded into a 96-well plate and cultured for 24 h. After removing the medium, an equal volume of serum-free DMEM was added, and the cells were cultured for another 24 h. The supernatant was then collected and centrifuged to remove cell debris. Subsequently, the concentration of secreted CSF1 was measured according to the instructions of the Human Macrophage Colony-Stimulating Factor (M-CSF) ELISA Kit (HM10372, Bioswamp Life Science Lab, China).

### Transwell assays

Transwell assays were performed to assess the ability of GBM cells and hybrids to recruit macrophages. Briefly, macrophages were placed in the upper chamber, while GBM cells or hybrids were placed in the lower chamber. After 48 h of incubation, non-migrated cells were scraped off using a cotton swab, and the cells at the bottom of the chamber were fixed with 4% formaldehyde for 10 min and stained with 0.5% crystal violet. Then, three random fields were photographed under an inverted microscope, and the cells were counted.

### Flow cytometry (FCM)

After the mice were euthanized, the collected intracranial tumor tissues were dissociated into single-cell suspensions using a papain dissociation kit (Worthington Biochemical, USA) according to the recommended protocol. For surface staining, the cells were incubated for 15 min with the antibody mixture in FACS buffer (PBS containing 0.1% BSA and 0.02% sodium azide). The Fixable Viability Dye eFluor™ 506 (eBioscience, USA) was used to exclude dead cells from the analysis. For cells requiring intracellular staining, the True-Nuclear™ Transcription Factor Buffer Set (eBioscience, USA) was used according to the staining protocol. Samples were acquired using a BD FACSymphony™ A5 Cell Analyzer, and the data were analyzed using FlowJo (version 10.8.1). During the analysis, myeloid cells were first selected using CD11b. Subsequently, F4/80 and CD206 were used to label total macrophages and M2-like macrophages, respectively. All antibodies used in the experiments are listed in Supplementary Table S2.

### m^6^A-methylated RNA immunoprecipitation qPCR (Me-RIP-qPCR)

According to the instructions provided by the Magna MeRIP™ m^6^A Kit (17-10499, Millipore), we first extracted RNA from cells using TRIzol reagent. The extracted RNA was then purified using the GenElute mRNA Miniprep Kit (MRN10, Sigma) and subsequently fragmented. The fragmented mRNA was divided into input and IP groups. The input group served as a control to observe the original expression levels of the samples. The IP group was incubated with magnetic beads coupled with either m^6^A antibodies or IgG antibodies. Following elution, the resulting products were analyzed by qPCR. The primers used in this study are listed in Supplementary Table S3.

### RIP-qPCR

We performed RIP-qPCR following the instructions of the EZ-Magna RIP™ RNA-Binding Protein Immunoprecipitation Kit (17-700, Millipore). Briefly, cell supernatants were lysed with RIP assay lysis buffer and then divided into input and IgG groups. The input and IgG groups were incubated overnight at 4 °C with the YTHDF2 antibody and IgG, respectively. Subsequently, the magnetic beads protein A/G were added and the mixture was incubated with shaking at 4 °C. After the unbound material was thoroughly washing off, the bound RNA was eluted and purified. Finally, qPCR was performed to analyze the RNA. The primers used in this experiment are also listed in Supplementary Table S3.

### mRNA stability assay

Cells were treated with 5 µg/ml actinomycin D (A9415, Sigma) to inhibit global mRNA transcription. Then, the cells were collected at the indicated time points post-treatment. Total RNA was extracted, and qPCR was performed to assess the expression levels of CSF1.

### Statistical analysis

The sample size for each group, including animal experiments (*n* = 5), was determined based on prior similar studies and experimental feasibility. Blinding was applied during the outcome assessment to minimize bias. Data analysis and visualization were performed by R software (v4.2.3) and GraphPad Prism (v9.0.0). The results are representative of three independent experiments and are presented as the mean ± SD. Student’s *t* test or one-way ANOVA (followed by Bonferroni post hoc correction) was utilized to compare continuous variables between two groups or more than two groups. All data are presented as the mean ± standard deviation (SD) of at least three independent experiments. All the statistical tests were bilateral, and a *P* value < 0.05 was considered to indicate statistically significant. ns not significant; *p < 0.05, **p < 0.01, ***p < 0.001.

## Results

### Identification of double-positive cells with GBM cells and MSCs markers

**Fig. 1:**
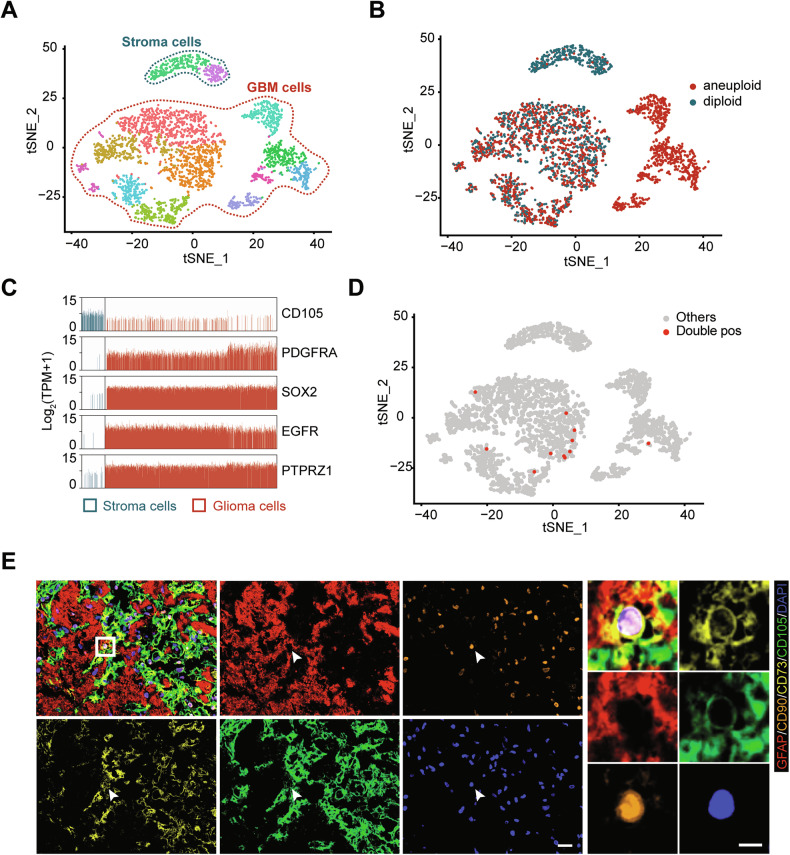
Identification of double-positive cells with GBM and MSCs markers. **A** t-SNE map displaying the clustering of 3459 cells. Each dot represents an individual cell, colored according to its cluster. **B** The CopyKat algorithm was used to distinguish between benign (diploid) and malignant (aneuploid) cells, presented in a t-SNE plot. **C** Bar plots illustrating the mRNA expression levels of the indicated markers, measured by log2-TPM (transcripts per million reads). Each bar represents a single cell, colored by the cell types identified in (**A**). **D** A t-SNE plot showing the distribution of double-positive (double-pos) cells, colored by cell types. **E** IF images showing the cell co-expressing GFAP (red), CD90 (orange), CD73 (yellow), and CD105 (green) in GBM specimens (GBM506). Scale bar: 20 μm (left), 5 μm (right).

Previous studies have demonstrated the fusion of BMSCs and GBM cells, yet whether GA-MSCs exhibit similar fusion remains unclear. We integrated single-cell RNA sequencing data of three GBM patients from a published study, which included 6337 cells after quality control [30]. To comprehensively understand the population structure, we employed t-distributed stochastic neighbor embedding (t-SNE) to create a two-dimensional map. Unsupervised clustering segregated cells into 17 clusters, which were subsequently annotated into seven cell types (Supplementary Fig. S1A). We then selected cells identified as GBM cells and stromal cells for further unsupervised clustering, and the results showed a clear distinction between the two cell types (Fig. 1A; Supplementary Fig. S1B–D). The “stromal cells” cluster, enriched with non-malignant cells, exhibited high expression of CD105, a marker commonly associated with mesenchymal cells. In contrast, the “GBM cells” cluster predominantly expressed markers such as PDGFR, SOX2, EGFR, and PTPRZ1, which are frequently observed in GBM cells but are rarely expressed in MSCs. (Fig. 1B, C). Cells co-expressing markers indicative of GA-MSCs (CD73, CD90, and CD105) and GBM cells (GFAP, EGFR, PTPRZ1, and SOX2) were identified as double-positive cells, with 11 instances observed among 3459 cells (Fig. 1D).

Additionally, we employed commonly utilized markers to identify GBM cells (GFAP) and MSCs (CD105, CD73, and CD90) in human GBM surgical specimens. We also provided hematoxylin and eosin (HE) staining images to support the glioma origin of the samples (Supplementary Fig. S1E). Subsequently, cells co-expressing markers from both cell populations were observed (Fig. 1E). These findings suggest the presence of hybrids formed by GA-MSCs and GBM cells in human GBM.

### GBM cells could fuse with GA-MSCs both in vitro and in vivo

**Fig. 2:**
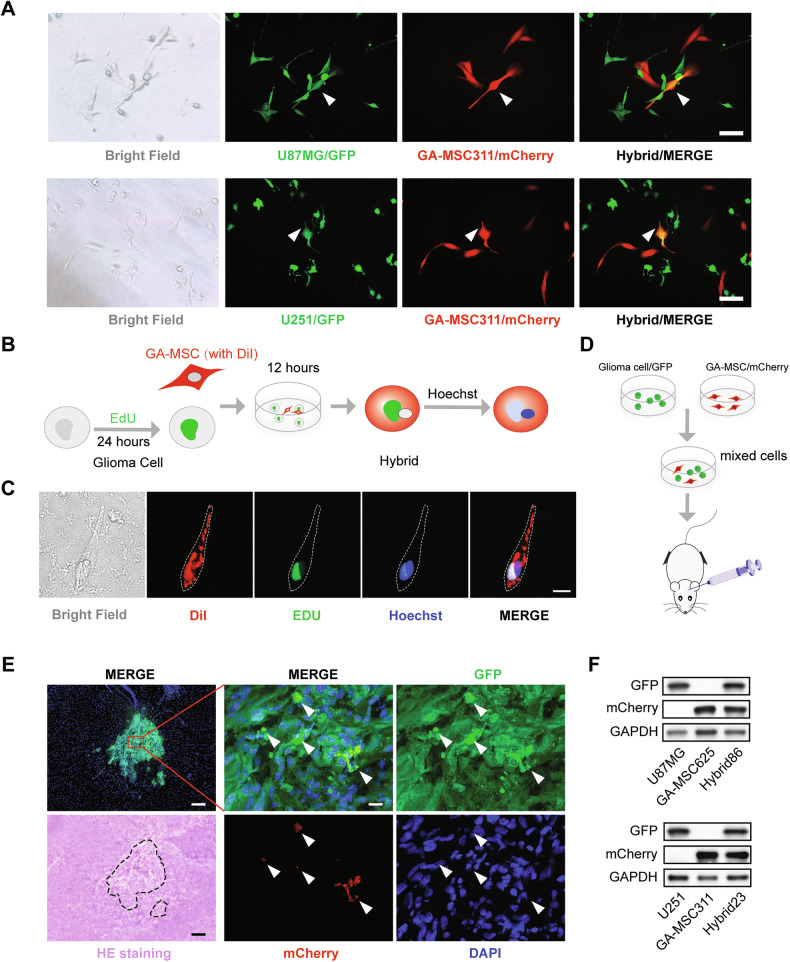
GBM cells could fuse with GA-MSCs in vitro and in vivo. **A** Bright field and fluorescence images of GA-MSC311-mCherry co-cultured with U87MG-GFP and U251-GFP. The white arrows indicate mCherry+/GFP+ hybrid cells. Scale bar, 100 μm. **B**, **C** Schematic and corresponding bright field and fluorescence images of the EDU labeling experiment in the co-culture model. Components from both cell types can be detected in the hybrids. Scale bar, 20 μm. **D** Schematic of the in vivo co-culture experiment with U87MG-GFP and GA-MSC-mCherry. **E** HE staining and fluorescence images of tumors in the in vivo co-culture mouse model, with white arrows indicating mCherry+/GFP+ cells. Scale bar: 200 μm (left), 20 μm (right). **F** Western blot analysis of GFP and mCherry expression in U87MG, U251, and hybrid cells.

To further investigate the fusion of GBM cells and GA-MSCs, we labeled GBM cells (U87MG and U251) with GFP and hygromycin B resistance, and GA-MSCs (GA-MSC311 and GA-MSC625) with mCherry and blasticidin resistance, and then established a co-culture system of these two cell types. In various pairwise combinations, we observed cells expressing both GFP and mCherry after 24 h of co-culture, referred to as hybrid cells (Fig. 2A; Supplementary Fig. S2A). In addition, we labeled the nuclei of GBM cells with EdU and GA-MSCs with CellTracker Red CM-Dil. After 12 h of co-culture, we stained the nuclei with blue Hoechst (Fig. 2B). The presence of cells with red membranes and green nuclei further confirmed the spontaneous fusion between the two cell types (Fig. 2C).

We utilized an intracranial xenograft model to investigate the fusion of GBM cells and GA-MSCs in vivo. Initially, we combined U87MG-GFP with GA-MSCs-mCherry and implanted these mixtures into mouse brains. After three weeks, tumors formed by GFP-expressing cells were observed in the mouse brain, while no cells expressing mCherry alone were found within the tumors. Importantly, some tumor cells co-expressed both GFP and mCherry, demonstrating the fusion capability of the two cell types in vivo (Fig. 2D, E).

Moreover, hybrid cells exhibiting dual drug resistance were obtained by treating the co-culture system with both hygromycin B and blasticidin. The co-expression of GFP and mCherry was confirmed by IF and Western blot (Supplementary Fig. S2B, C; Fig. 2F). We adopted a nomenclature for the hybrid cells based on the first digit of the parental GBM cell and GA-MSC; for instance, Hybrid83 denotes a fusion of U87MG and GA-MSC311.

### The hybrids showed reduced levels of m^6^A mediated by FTO, contributing to their enhanced growth in vivo

**Fig. 3:**
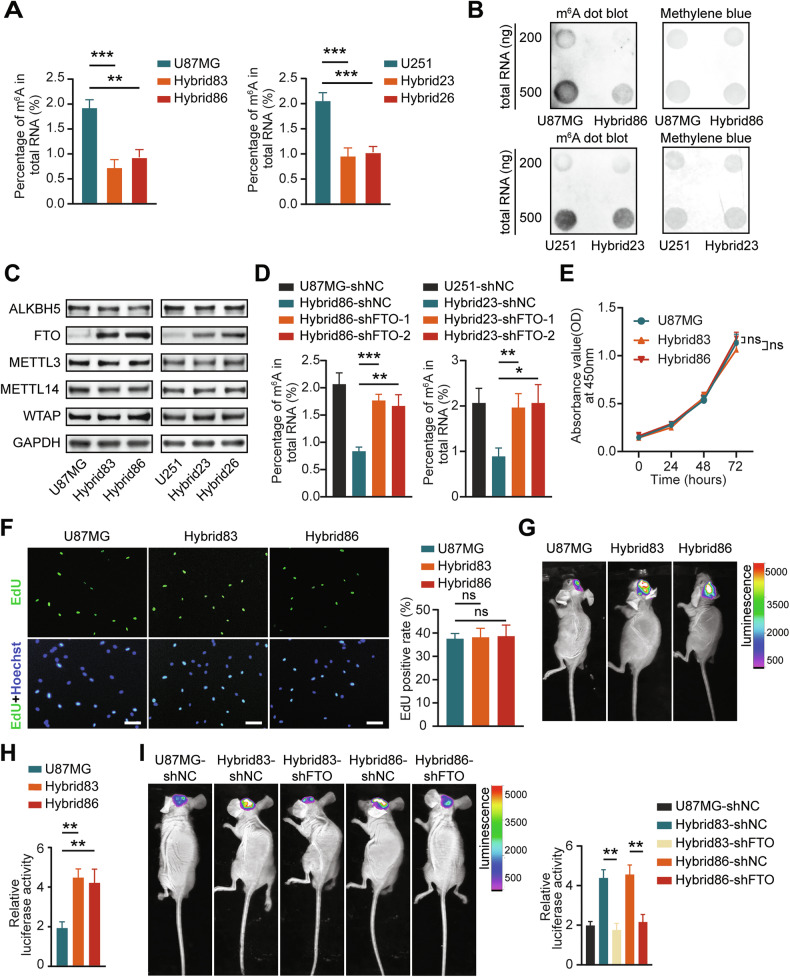
The hybrids show reduced levels of m^6^A mediated by FTO, contributing to their enhanced growth in vivo. **A** Detection of m^6^A levels in GBM cells and hybrids by measuring the percentage of m^6^A in total RNA using the m^6^A RNA methylation quantification kit. **B** Dot blot analysis of m^6^A levels in GBM cells and hybrids. **C** Western blotting analysis of ALKBH5, FTO, METTL3, METTL14, and WTAP expression in GBM cells and hybrids. **D** Measurement of m^6^A levels in hybrids following FTO knockdown using the m^6^A RNA methylation quantification kit. **E** Assessment of proliferation in U87MG, Hybrid83 and Hybrid86 using the CCK-8 assay. **F** Representative images and statistical results of EdU assays demonstrating the proliferation of U87MG, Hybrid83 and Hybrid86. Scale bar: 100 μm. **G**–**I** Representative bioluminescence images and statistical results 3 weeks after the implantation of cells from each group.

m^6^A modification is the most prevalent RNA modification of eukaryotic mRNAs and plays a crucial role in the progression of various tumors, including glioma [31]. However, the change in m^6^A modification following cell fusion in tumors remains unknown. To investigate the changes in m^6^A modification after GBM cells were fused with GA-MSCs, we examined the m^6^A modification levels in hybrids and the parental GBM cells using m^6^A detection assay kits and dot blot analysis. As shown in Fig. 3A, B and Supplementary Fig. S3A, the hybrids exhibited significant lower m^6^A levels than their parental GBM cells. While we also analyzed the m^6^A levels in hybrids relative to their parental GA-MSCs, no consistent patterns were observed (Supplementary Fig. S3B, C).

Methyltransferases (writers) and demethylases (erasers) are direct regulatory factors that influence m^6^A modification [32]. Thus, we evaluated the expression levels of common m^6^A writers (METTL3, METTL14, and WTAP) and erasers (FTO and ALKBH5). The results showed that the expression of FTO in U87MG and U251 was consistently elevated after fusion with GA-MSCs, while other enzymes showed no significant changes (Fig. 3C).

To clarify the role of FTO in this m^6^A modification alteration, we knocked down FTO with two distinct shRNAs in U87MG, U251 and hybrids. The efficiency was confirmed by qPCR and Western blot analysis (Supplementary Fig. S3D, E). We found that the m^6^A levels of hybrids transfected with shFTO were significantly higher than those of hybrids transfected with shNC (Fig. 3D; Supplementary Fig. S3F), suggesting that knocking down FTO can rescue the phenotype of decreased m^6^A levels in hybrids. These findings strongly suggest that FTO mediates the changes in m^6^A levels in hybrids.

We also characterized the growth of hybrids, and the results of both CCK8 and EdU assays demonstrated that the proliferation rate of hybrids in vitro was comparable to their parental GBM cells (Fig. 3E, F; Supplementary Fig. S3G, H). However, in the mouse intracranial xenograft model, tumors originating from Hybrid83 and Hybrid86 were larger than those from U87MG (Fig. 3G, H, Supplementary Fig. S3I). Knocking down FTO in Hybrid83 and Hybrid86 partially rescued their increased growth rates in vivo (Fig. 3I). These results suggest that hybrids have a growth advantage only in vivo, which is related to their lower m^6^A levels.

### CSF1 exhibits high expression and decreased m^6^A modification levels in hybrids

**Fig. 4:**
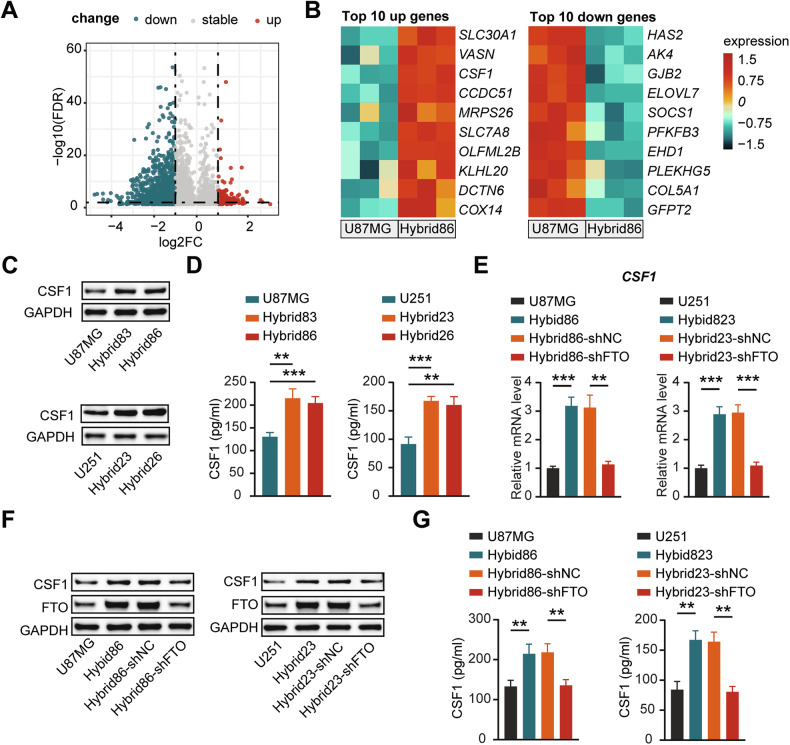
CSF1 exhibits high expression and decreased m^6^A modification levels in hybrids. **A** Volcano plot showing the distribution of differences in m^6^A modification levels between U87MG and Hybrid86. **B** Heatmap displaying the top 10 genes upregulation or downregulation in expression among those with decreased m^6^A levels in Hybrid86 compared to U87MG. **C** Western blotting analysis of CSF1 protein expression in GBM cells and hybrids. **D** Secreted CSF1 in the supernatants of GBM cells and hybrids were detected by ELISA. Examination of the effect of FTO knockdown on CSF1 expression in hybrids using qPCR (**E**), western blotting (**F**), and ELISA (**G**).

To explore the mechanism in hybrid that promote tumor growth due to decreased m^6^A levels, we performed m^6^A -seq to map the m^6^A methylomes of U87MG and Hybrid86. The data indicated that m^6^A sites were highly enriched in the consensus GGAC motif in both U87MG and hybrid86, especially in the 3’UTR and coding sequence region (Supplementary Fig. S4A, B). As shown in Fig. 4A, a total of 1826 m^6^A peaks were significantly altered (FDR < 0.05 and log 2 |fold change (FC)| >1), of which 162 were hypermethylated and 1664 were hypomethylated. To characterize the functional role of m^6^A modification, we performed Kyoto Encyclopedia of Genes and Genomes (KEGG) pathway enrichment analysis of genes with different m^6^A peaks. The results revealed several tumor-related pathways among the top significant pathways, such as “pathways in cancer”, “prostate cancer” and “chronic myeloid leukemia”, further confirming the important role of m^6^A modification in cancer progression (Supplementary Fig. S4C). We also conducted KEGG enrichment analysis of the differentially expressed genes, and one of the top significant enriched pathways was “cytokine−cytokine receptor interaction”, indicating that fusion with GA-MSCs may promote tumor progression through cytokine-mediated interactions with the TME (Supplementary Fig. S4D).

Since hybrids grow more vigorously in vivo in relation to their low m^6^A levels, we focused on genes with reduced m^6^A modification. The top 10 up/downregulated genes among the genes that underwent demethylation are shown in Fig. 4B. We selected 8 genes related to tumor progression and validated their expression by qPCR in different pairs of GBM cells and hybrids. CSF1 exhibited the most significant and consistent change (Supplementary Fig. S4E). Furthermore, we validated that the expression of CSF1 was higher in hybrids than in corresponding GBM cells by western blot analysis and ELISA (Fig. 4C, D). To characterize the correlation between CSF1 expression and m^6^A levels, we examined CSF1 expression in Hybrid86 and Hybrids23 after FTO knockdown and found that FTO knockdown significantly downregulated CSF1 levels in hybrids (Fig. 4E–G). This result implies that the increased expression of CSF1 may be related to its reduced m^6^A levels.

### Hybrids have an enhanced ability to induce macrophage recruitment and M2-like polarization

CSF1 is well-known as a cytokine that modulates macrophages, which play an active role in the GBM TME and can promote tumor development through differentiation toward the M2 phenotype [33]. CSF1 is essential for macrophage survival and differentiation, and blocking the CSF1/CSF1 receptor has been proven to significantly inhibit GBM progression [34]. We thus hypothesize that hybrids may activate macrophages through CSF1, thereby promoting tumor growth in vivo.

**Fig. 5:**
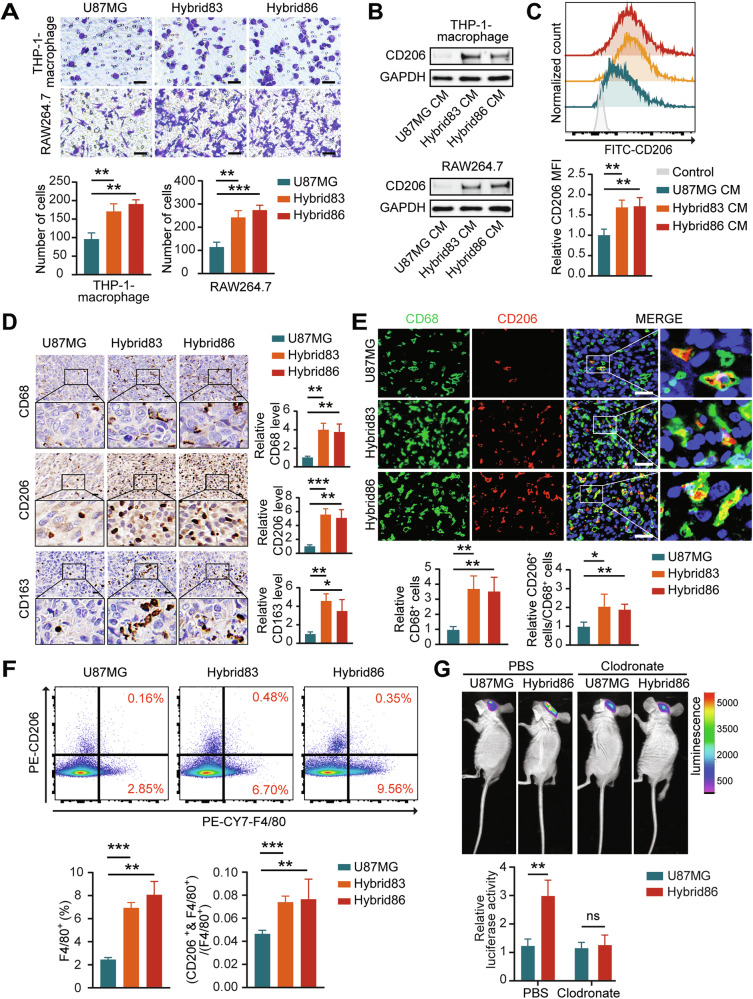
Hybrids have an enhanced ability to induce macrophage recruitment and M2 polarization. **A** Representative images and statistical results of the macrophage transwell assay. Scale bar:50 μm. **B** Western blot analysis of CD206 expression in macrophages treated with CM supernatant from GBM cells or hybrids for 48 h. **C** FCM measurement of CD206 levels in THP-1-macrophages from each group. **D** Representative images and statistical results of IHC staining for CD68, CD206 and CD163 levels in the different groups. Scale bar: 20 μm. **E** Representative images and statistical results of IF staining showing the distribution and proportion of CD68 and CD206 positive cells in the different groups. Scale bar: 40 μm. **F** Flow cytometry analysis of the proportion of F4/80+ and F4/80+/CD206+ positive cells among CD11b+ cells in different groups. **G** Representative bioluminescence images and statistical results 3 weeks after cell implantation in mice treated with clodronate or PBS.

To investigate the effects of the hybrids on macrophages, we examined the ability of U87MG, Hybrids83 and Hybrids86 to recruit macrophages by transwell assays. The results indicated that the hybrids exhibited an enhanced ability to recruit both human THP-1-macrophage and mouse RAW264.7 macrophage (Fig. 5A). Besides, the levels of M2 marker CD206 in both human and mouse macrophages were higher after treatment with culture medium (CM) supernatant from Hybrid83 or Hybrid86 than with CM from U87MG (Fig. 5B, C).

We also explored the ability of hybrids to activate macrophages under in vivo conditions. Total macrophages were labeled with the recognized marker CD68 (for IHC and IF assays) or F4/80 (for FCM), and M2-like macrophages were labeled with CD206 and CD163. The results showed that both total and M2-like macrophages were more abundant in tumors formed by Hybrid83 and Hybrid86 than in those formed by U87MG, and the proportion of M2-like macrophages among total macrophages was also higher in tumors formed by hybrids (Fig. 5D–F).

To determine the role of macrophages in causing differences in growth between hybrids and GBM cells in vivo, we injected our mouse intracranial xenograft model with the macrophage-depleting agent clodronate. The IF results indicated that CD68- and CD206-positive cells were sparse in both the clodronate-treated U87MG and Hybrid86 groups. Furthermore, no significant differences were detected in CD68 expression levels or the CD206/CD68 ratio between the two groups (Supplementary Fig. S5A). There was no significant difference in tumor size between tumors originated from U87MG and Hybrid86 under macrophage depletion conditions, confirming that macrophages were the central factor responsible for the differences in tumor growth (Fig. 5G).

### Inhibition of FTO impairs the effect of the hybrid on macrophages

**Fig. 6:**
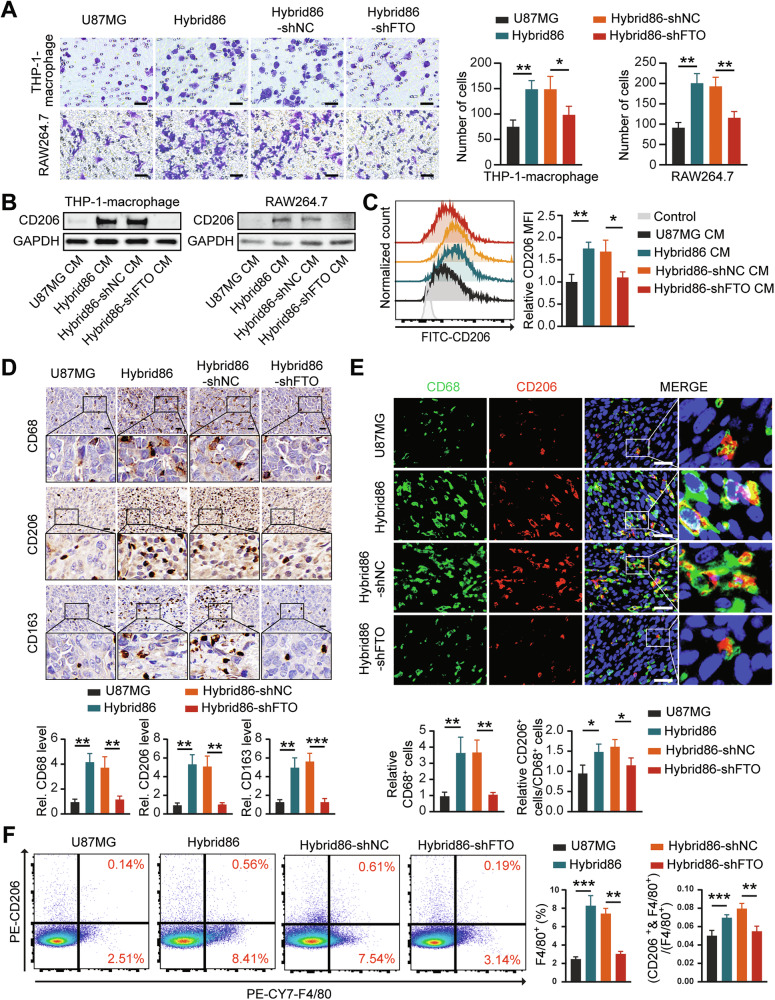
Inhibition of FTO impairs the effect of the hybrid on macrophages. **A** Representative images and statistical results of the macrophage transwell assay. Scale bar: 50 μm. **B** Western blot analysis of CD206 expression in macrophages treated with CM from different cells for 48 h. **C** FCM evaluation of CD206 levels in THP-1-macrophages in each group. **D** Representative images and statistical results of IHC staining for CD68, CD206 and CD163 levels in the different groups. Scale bar: 20 μm. **E** Representative images and statistical results of IF staining showing the distribution and proportion of CD68 and CD206 positive cells in different groups. Scale bar: 40 μm. **F** Flow cytometry analysis of the proportions of F4/80^+^ and F4/80^+^/CD206^+^ cells among CD11b^+^ cells in different groups.

To explore the relationship between the m^6^A levels of hybrids and their ability to stimulate macrophages, we transfected hybrids with shFTO and found that inhibiting FTO in hybrids reduced their ability to recruit macrophages (Fig. 6A). Furthermore, knockdown of FTO in hybrids significantly diminished their ability to induce macrophage M2-like polarization (Fig. 6B, C).

We then investigated the effect of knocking down FTO in hybrids on macrophages in the mouse model. Compared to tumors derived from Hybrid86-shNC, tumors formed from Hybrid86-shFTO had lower CSF1 expression and fewer total and M2-like macrophages (Fig. 6D). Compared to tumors originating from Hybrid86-shNC, we observed lower CSF1 expression, less macrophage infiltration, and a lower M2-like/total macrophages rate in the Hybrid86-shFTO group (Supplementary Fig. S5B, Fig. 6E, F). The above results demonstrate that CSF1 expression is regulated by FTO in hybrids, which in turn promotes M2-like polarization of recruited macrophages.

### FTO regulates CSF1 expression in an m^6^A-YTHDF2-dependent manner

**Fig. 7:**
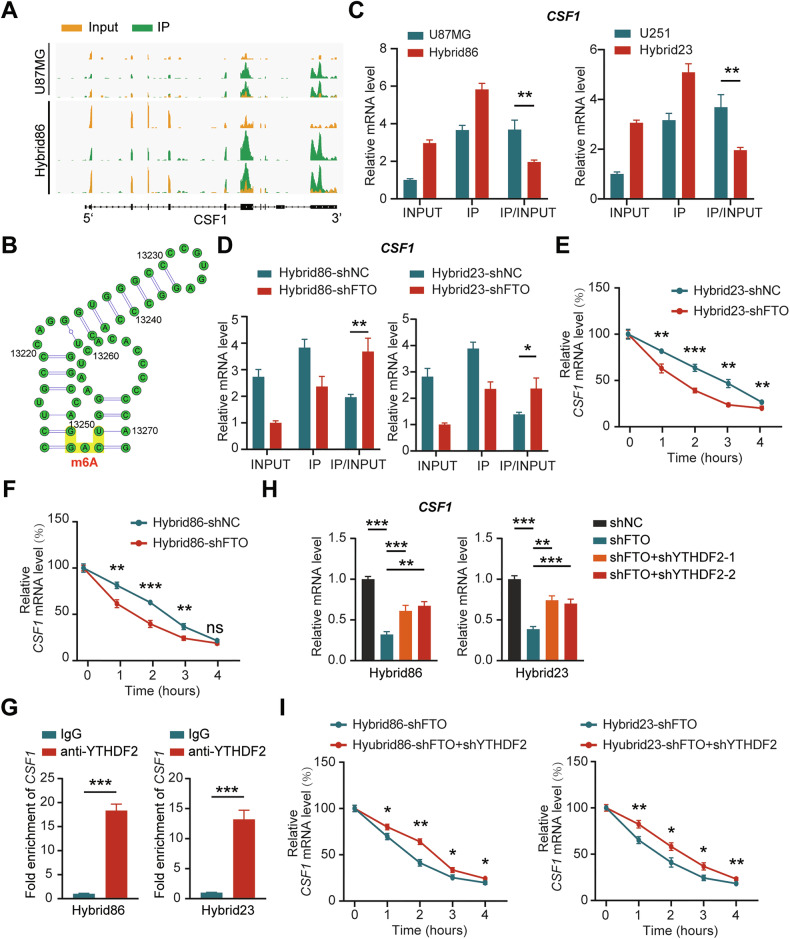
FTO regulates CSF1 expression in an m^6^A-YTHDF2-dependent manner. **A** IGV analysis illustrating the m^6^A modification levels of CSF1 mRNA in U87MG and Hybrid86 based on Me-RIP-seq data. **B** Schematic diagram of the predicted m^6^A modification site in CSF1 mRNA as identified by SRAMP. **C** Detection of m^6^A modification levels of CSF1 mRNA in GBM cells and hybrids using the MeRIP m^6^A Kit. **D** Detection of the m^6^A modification levels of CSF1 mRNA in control and FTO knockdown hybrids using the MeRIP m^6^A Kit. **E**, **F** qPCR analysis of CSF1 mRNA levels in control and FTO knockdown hybrids after actinomycin D treatment for 0, 1, 2, 3, and 4 h. **G** YTHDF2 was immunoprecipitated and then subjected to qPCR to assess CSF1 transcript levels. **H** qPCR analysis of CSF1 mRNA levels in hybrids with FTO knockdown alone and with both FTO and YTHDF2 knockdown. **I** qPCR analysis of CSF1 mRNA levels in hybrids with FTO knockdown alone and with both FTO and YTHDF2 knockdown after actinomycin D treatment for 0, 1, 2, 3, or 4 h.

Our m^6^A-seq data showed that the m^6^A level of CSF1 mRNA was lower in Hybrid86 than in U87MG (Fig. 7A). The site with the highest confidence of CSF1 mRNA m^6^A modification was predicted by SRAMP, and we designed a primer for this site to detect CSF1 mRNA m^6^A levels by Me-RIP-qPCR (Supplementary Fig. S6A; Fig. 7B) [35]. The results showed that CSF1 mRNA m^6^A levels in the hybrids were consistently lower than those in their parental GBM cells, and further data showed that knockdown of FTO could partially reverse this alteration (Fig. 7C, D; Supplementary Fig. S6B, C). In addition, we observed that CSF1 mRNA degradation was increased upon FTO knockdown, confirming that the upregulation of CSF1 expression in hybrids may result from increased stability of its mRNA due to reduced m^6^A modification. (Fig. 7E, F; Supplementary Fig. S6D).

m^6^A modification performs biological functions through binding proteins called “readers”, which influence mRNA translation efficiency and stability. YTHDF2 is a prominent “reader” that facilitates mRNA degradation and has been implicated in targeting CSF1 mRNA [36]. We thus hypothesized that YTHDF2 is the “reader” of the m^6^A modification of CSF1 mRNA. To test this hypothesis, we conducted RNA RIP assays, revealing a robust interaction between YTHDF2 and CSF1 mRNA (Fig. 7G). Additionally, we knocked down YTHDF2 in FTO-reduced hybrids (as verified by qPCR and WB), which resulted in a partial recovery of CSF1 expression (Supplementary Fig. S6E, F; Fig. 7H). YTHDF2 knockdown could also rescue the decrease in CSF1 mRNA stability under FTO knockdown conditions, confirming that YTHDF2 contributes to CSF1 m^6^A modification, even though we did not observe differences in its expression between hybrids and corresponding GBM cells (Fig. 7I; Supplementary Fig. S6G). These results support the notion that FTO regulates CSF1 mRNA levels in an m^6^A -YTHDF2-dependent manner.

**Fig. 8:**
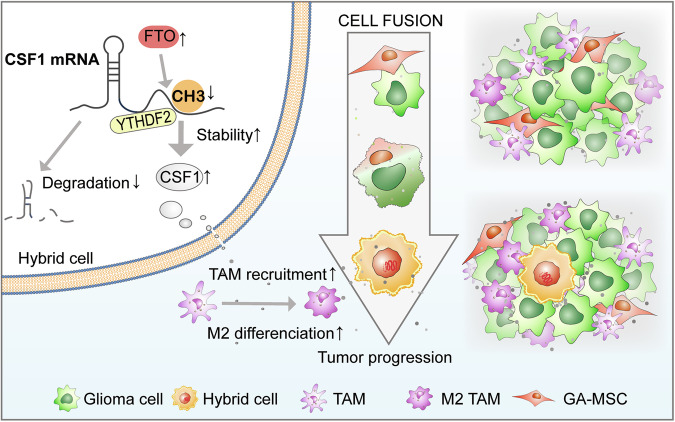
Schematic diagram illustrating the fusion of GA-MSCs with glioma cells promoting macrophage recruitment and M2 polarization via FTO-mediated CSF1 secretion in the TME.

Overall, these findings indicate that the fusion of GA-MSCs with glioma cells promotes macrophage recruitment and M2-like polarization through FTO-mediated CSF1 secretion within the TME (Fig. 8).

## Discussion

GBM is a highly malignant tumor with a poor prognosis, and its maintenance and progression are largely attributed to its dynamic interactions with the TME. Among the immune cells within the GBM TME, macrophages, particularly the M2 subtype, play a crucial role in supporting and driving tumor progression. Previous studies have shown that GBM with higher GA-MSCs abundance exhibits increased infiltration of M2-like macrophages [23, 37, 38]. However, the mechanism underlying the crosstalk between GA-MSCs and macrophages in the GBM TME remains unclear. Our research confirmed that GBM cells can spontaneously fuse with GA-MSCs, forming new hybrids that regulate macrophage behavior through CSF1.

Our study confirms that GBM cells can spontaneously fuse with GA-MSCs. Notably, the newly formed hybrids exhibited a proliferation advantage over GBM cells exclusively in vivo, with comparable growth rates observed in vitro. To elucidate this phenomenon, we conducted m^6^A -seq followed by validation experiments, which highlighted the pivotal role of macrophages in enhancing the in vivo proliferative phenotype of hybrids. Previous studies have extensively documented the critical involvement of GBM–macrophage interactions in tumor progression, identifying them as a promising therapeutic target [28, 39]. Our findings indicate that the growth advantage of hybrids is predominantly mediated by macrophage regulation in the TME, rather than by intrinsic alterations in their proliferative capacity. Mechanistically, we found that the m^6^A demethylase FTO is upregulated after the fusion of GBM cells and GA-MSCs, leading to increased CSF1 secretion through an m^6^A -YTHDF2-dependent mechanism.

Cell fusion enhances tumor heterogeneity, which in turn drives tumor evolution and progression [40]. The impact of cell fusion on tumor progression might exceed our expectations. In a breast cancer mouse model, the cell fusion rate was about 6% without treatment, and rose to approximately 12% following chemotherapy [41]. Similarly, another study reported that the cell fusion rate in an ovarian cancer animal model could reach approximately 4% [42]. More importantly, cell fusion may produce more malignant tumor cells with greater adaptability, which are more likely to survive under competitive stress. Consequently, hybrid cells may be more likely to expand their progeny, further acting as a hidden force driving tumor development.

The process and mechanisms of two cells becoming one are complex and varied, including processes such as cell fusion and phagocytosis. Here, we use the term “fusion” broadly to describe the merging of two cells into a hybrid cell without specifying the exact mechanisms of hybridization. Instead, we focused on the biological properties and functions of the newly formed hybrid cells. Perhaps due to their multipotent “stem-like” potential, MSCs often observed to fuse with cancer cells, making them favorable candidates for studying cell fusion [43]. However, MSCs lack a unique specific marker, making it challenging to study MSC fusion with other cells in vivo. To improve accuracy, we employed multiple marker co-expression to label GA-MSCs, though this approach is not entirely rigorous. Utilizing exogenous markers like GFP may provide a more effective means to label targeted MSCs. However, it still cannot precisely track the fusion process, as newly formed hybrids may unpredictably lose segments of the parental cell components [44]. These challenges have limited our understanding of cell fusion, even though this phenomenon has been recognized for more than a century. Conversely, cells containing major components from two different cell types can be considered outcomes of cell fusion, providing a method to identify hybrid cells, although this approach may not capture all instances.

Cell fusion leads to unpredictable genetic material remodeling. Although hybrids generally exhibit characteristics of the parental cells, their transcriptomes display significant uncertainty, and even less is known about the changes in their RNA modifications [15, 45]. The role of FTO in gliomas remains controversial, with different studies presenting conflicting results [46, 47]. Nonetheless, its role as an m^6^A demethylase that decreases m^6^A levels on target RNAs is well established [32]. Our research demonstrated that the fusion of GBM cells and GA-MSCs leads to FTO-mediated changes in CSF1 mRNA m^6^A modification, consequently remodeling the macrophage-related TME through CSF1.

The role of macrophages in the maintenance and progression of gliomas has been well-established by substantial evidence [34, 48]. GA-MSCs are also associated with poor prognosis in gliomas, with indications suggesting a potential link between these two cell types [9]. Macrophages are most abundant infiltrated in the mesenchymal subtype of glioblastoma, which is also the subtype with the worst prognosis and the highest abundance of GA-MSCs [49, 50]. A recent study grouped GBM patients based on a gene set associated with GA-MSCs, and revealed that those with elevated GA-MSCs levels exhibited increased macrophage infiltration [38]. In one of our previous studies, we initiated tumors by separately injecting GBM cells or a mixture of GBM cells and GA-MSCs into the mouse brain. From this, we identified GA-MSC-related genes and established a prognostic index, which showed increased infiltration of M2-type macrophages in patients with higher GA-MSC relevance [23]. These data highlight the close interaction between GA-MSCs and macrophages but have not elucidated the underlying mechanisms involved.

Our findings revealed that the fusion of GBM cells with GA-MSCs recruits and educates macrophages through CSF1 secretion, elucidating the pivotal role of cell fusion in this regulatory axis. In recent years, with increasing recognition of the role of cell fusion in tumor progression, targeting cell fusion for tumor elimination has emerged as a novel and promising approach [51].

There are certain limitations in our study, with a prominent one being the inadequate distinction and detailed analysis of the macrophage subpopulations involved. The macrophages in the GBM TME primarily consist of brain-resident microglia and bone marrow-derived macrophage. Traditionally, activated macrophages are categorized into M1 and M2 subtypes, with M1 macrophages mediating pro-inflammatory, anti-tumor responses, while M2 macrophages are associated with anti-inflammatory responses and tumor promotion [52].

Despite their distinct origins, macrophages within the GBM TME share overlapping markers and functions, such as responsiveness to CSF1 stimulation and susceptibility to clodronate-induced depletion [4, 34, 53]. Thus, many studies on GBM-associated macrophages do not strictly distinguish their origins and instead refer to the overall macrophage population. However, emerging evidence suggests that macrophages of different origins may exhibit spatial and functional heterogeneity, with potential origin-specific markers that can distinguish them [48, 53, 54]. In our study, the available data were insufficient to delineate the specific contributions of macrophages from distinct origins to the observed phenotypes. Therefore, we primarily focused on the impact of hybrids on macrophages as a bulk population. Additionally, although the M1/M2 classification is widely employed in tumor-related research, particularly those investigating the CSF1/CSF1R axis [55–58]. Growing evidence indicates that such a binary classification oversimplifies macrophage heterogeneity and plasticity [59, 60]. Notably, the classical M2 marker CD206 has been reported to exhibit pro-inflammatory functions in certain contexts, underscoring the complexity of macrophages in the TME [61, 62]. Our study mainly addresses the overall macrophage changes driven by increased CSF1 secretion from hybrids and their influence on tumor progression, without delving into a detailed characterization of macrophage subtypes. We will conduct further investigations on these aspects in future studies.

## Conclusions

In summary, our study confirmed the occurrence of fusion between GBM cells and GA-MSCs, demonstrating that the newly generated hybrids can promote GBM progression by regulating macrophages via the FTO/ m^6^A /CSF1 axis. This discovery sheds light on a new mechanism for intercellular communication within the GBM TME and offers novel insights into GBM therapeutic strategies.

## Supplementary information

Table S1-S3

Additional files

## Data Availability

The scRNA-seq data analyzed in this study can be accessed at https://www.ncbi.nlm.nih.gov/geo/query/acc.cgi?acc=GSE131928. The m^6^A -seq data generated and analyzed in this study is temporarily unavailable to the public due to confidentiality requirements for ongoing experiments. The datasets generated during and/or analyzed during the current study are available from the corresponding author on reasonable request.
