# Supplementary material for: Fusion of glioma-associated mesenchymal stem/stromal cells with glioma cells promotes macrophage recruitment and M2 polarization via m6A modification of CSF1
Source: Cell Death Dis. 2025 Apr 26;16(1):345. doi: 10.1038/s41419-025-07678-x (PMC12033374; doi:10.1038/s41419-025-07678-x)
Supplement: Supplementary file 1 — Table S1-S3 [file 41419_2025_7678_MOESM1_ESM.docx]

Table S1. Clinical information of the GBM patients.

| Number | Gender | Age | Grade | Histology | IDH |
| --- | --- | --- | --- | --- | --- |
| GBM311 | Male | 59 | IV | Glioblastoma | WT |
| GBM625 | Female | 63 | IV | Glioblastoma | WT |
| GBM506 | Female | 61 | IV | Glioblastoma | WT |
| MGH124^*^ | Female | 68 | IV | Glioblastoma | WT |
| MGH125^*^ | Male | 76 | IV | Glioblastoma | WT |
| MGH143^*^ | Male | 62 | IV | Glioblastoma | WT |

* Patients from the GSE131928 dataset for single-cell analysis.

Table S2. List of antibodies used in this study.

| Antibody | Manufacturer | Cat.No. | Host | Application |
| --- | --- | --- | --- | --- |
| Anti-GFAP | Proteintech | 16825-1-AP | Rabbit | IF |
| Anti-CD105 | Proteintech | 67075-1-Ig | Mouse | IF |
| Anti-CD90 | Proteintech | 66766-1-Ig | Mouse | IF |
| Anti-CD73 | Servicebio | GB115174 | Rabbit | IF |
| Anti-GFP | Proteintech | 50430-2-AP | Rabbit | WB |
| Anti-mCherry | Proteintech | 26765-1-AP | Rabbit | WB |
| Anti-GAPDH | Antgene | Ant425 | Rabbit | WB |
| Anti-ALKBH5 | Abcam | 195377 | Rabbit | WB |
| Anti-FTO | Abcam | 126605 | Rabbit | WB, IHC |
| Anti-METTL3 | Abcam | 195352 | Rabbit | WB |
| Anti-METTL14 | Abcam | 309096 | Rabbit | WB |
| Anti-WTAP | Immunoway | YT4908 | Rabbit | WB |
| Anti-CSF1 | Abcam | 233387 | Rabbit | WB, IHC |
| Anti-CD206 | Proteintech | 18704-1-AP | Rabbit | WB |
| Anti-CD206 | Abcam | 64693 | Rabbit | IHC, IF |
| Anti-CD68 | Abcam | 283654 | Rabbit | IHC, IF |
| Anti-CD163 | Abcam | 182422 | Rabbit | IHC |
| Anti-CD206-FITC | BD Biosciences | 551135 | Mouse | FCM |
| Anti-CD11b-PerCP-Cy5.5 | BD Biosciences | 550993 | Rat | FCM |
| Anti-F4/80- PE-Cy7 | Thermo Fisher Scientific | 25-4801-82 | Rat | FCM |
| Anti-CD206- PE- | Thermo Fisher Scientific | 12-2061-82 | Rat | FCM |
| Anti-YTHDF2 | Abcam | 220163 | Rabbit | WB, RIP |
| HRP conjugated Goat Anti-Mouse IgG | Servicebio | GB23301 | Goat | WB, IHC, IF |
| HRP conjugated Goat Anti-Rabbit IgG | Servicebio | GB23303 | Goat | WB, IHC, IF |

Table S3. Details of all primer sequences.

| Gene | Sequence | Application |
| --- | --- | --- |
| GAPDH | F: 5′- GGAAGCTTGTCATCAATGGAAATC -3′  R: 5′- TGATGACCCTTTTGGCTCCC -3′ | qPCR |
| FTO | F: 5′- CTTGACTGCCATCCTTGCCT -3′  R: 5′- CTAGGGTTTTGCTTCCAGAAGC -3′ | qPCR |
| CSF1 | F: 5′- CTGCGTCCGAACTTTCTATGAG -3′  R: 5′- CACATCTTGGCTGGAGCATTC -3′ | qPCR, mRNA stability assay |
| SLC30A1 | F: 5′- GTGGCCAATACCAGCAACTC -3′  R: 5′- TCTGGGGTTTTCTGGGTCTG -3′ | qPCR |
| VASN | F: 5′- TCTCACCTATCGCAACCTATCG -3′  R: 5′- CAGACGGAGTAAGTGGCGTT -3′ | qPCR |
| SLC7A8 | F: 5′- TTGCCAATGTCGCTTATGTCA -3′  R: 5′- AGAGACCCATTAACTCCTCCAAA -3′ | qPCR |
| HAS2 | F: 5′- GCCTCATCTGTGGAGATGGT -3′  R: 5′- TCCCAGAGGTCCACTAATGC -3′ | qPCR |
| AK4 | F: 5′- TGGATTCACCCTCCTAGCGGAA -3′  R: 5′- CTGTCTTAGCCTGGCAGCAACT -3′ | qPCR |
| SOCS1 | F: 5′- TTTTCGCCCTTAGCGTGAAGA -3′  R: 5′- GAGGCAGTCGAAGCTCTCG -3′ | qPCR |
| PFKFB3 | F: 5′- GTGCCTTAGCTGCCTTGAGA -3′  R: 5′- CCGACTCGATGAAAAACGCC -3′ | qPCR |
| YTHDF2 | F: 5′- TACATCAAAAGGATGGATTAAACG -3′  R: 5′- CTGTCCATAAGAAGTTAAGTAGGGC -3′ | qPCR |
| CSF1 | F: 5′- TCTGGAGAGGCCAGTGAGAT -3′  R: 5′- GGCAGATGGATGGTCTGTCT -3′ | Me-RIP-qPCR |
| CSF1 | F: 5′- GACCCAGCAACTTCCTCTCAG -3′  R: 5′- GGCTTCTGGAAAGCTGTGG -3′ | RIP-qPCR |
