# Supplementary material for: Fusion of glioma-associated mesenchymal stem/stromal cells with glioma cells promotes macrophage recruitment and M2 polarization via m6A modification of CSF1
Source: Cell Death Dis. 2025 Apr 26;16(1):345. doi: 10.1038/s41419-025-07678-x (PMC12033374; doi:10.1038/s41419-025-07678-x)

Additional file 1: Figure S1-S6

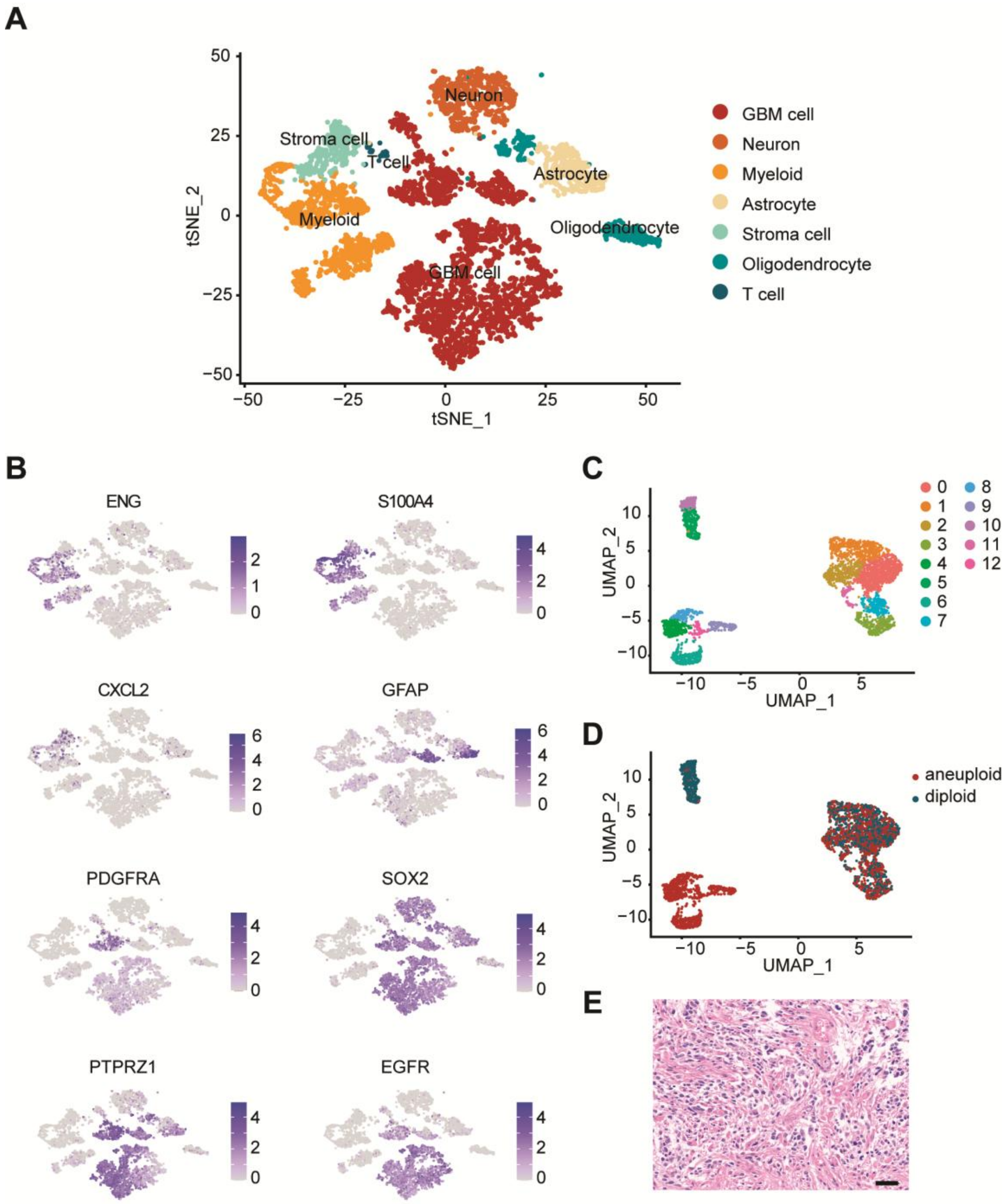

Fig. S1 GBM single-cell data and sample information. (A) t-SNE plot displaying the clustering of 6,337 cells. Each dot represents an individual cell, colored according to its cluster. (B) t-SNE plot showing the expression of stromal cell markers and GBM cell markers. (C) UMAP plot illustrating the clustering of 3,459 cells identified as stromal cells and GBM cells. (D) UMAP plot generated using the CopyKat algorithm

to distinguish between benign (diploid) and malignant (aneuploid) cells. (E) HE-stained image of the GBM sample. Scale bar: 50 μm.

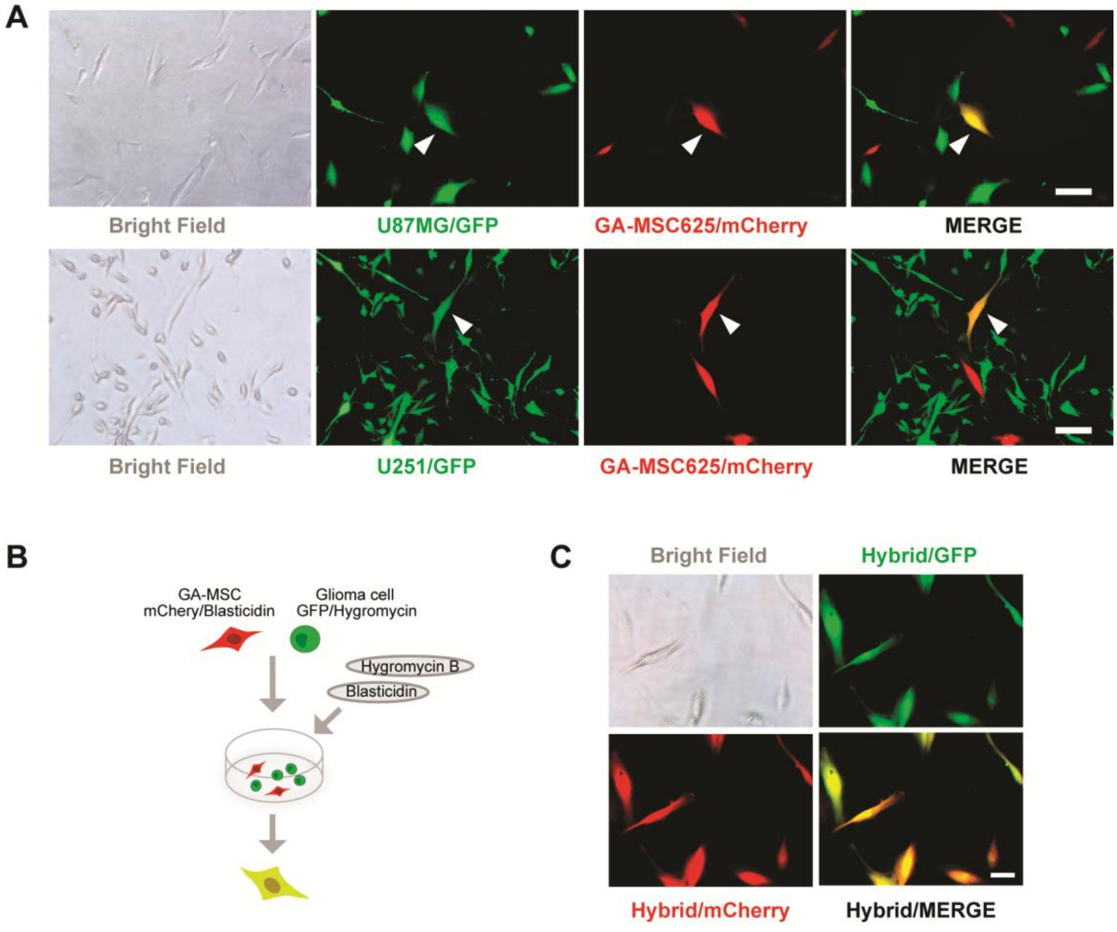

Fig. S2 GBM cells could fuse with GA-MSCs *in vitro*. (A) Bright field and fluorescence images of GA-MSC625-mCherry co-cultured with U87MG-GFP and U251-GFP. The white arrows indicate mCherry+/GFP+ hybrid cells. Scale bar, 100 μm. (B) Screening for cells expressing resistance to two drugs within the co-culture system to obtain hybrids. (C) Representative IF images showing the expression of GFP and mCherry in the obtained hybrids. Scale bar: 50 μm.

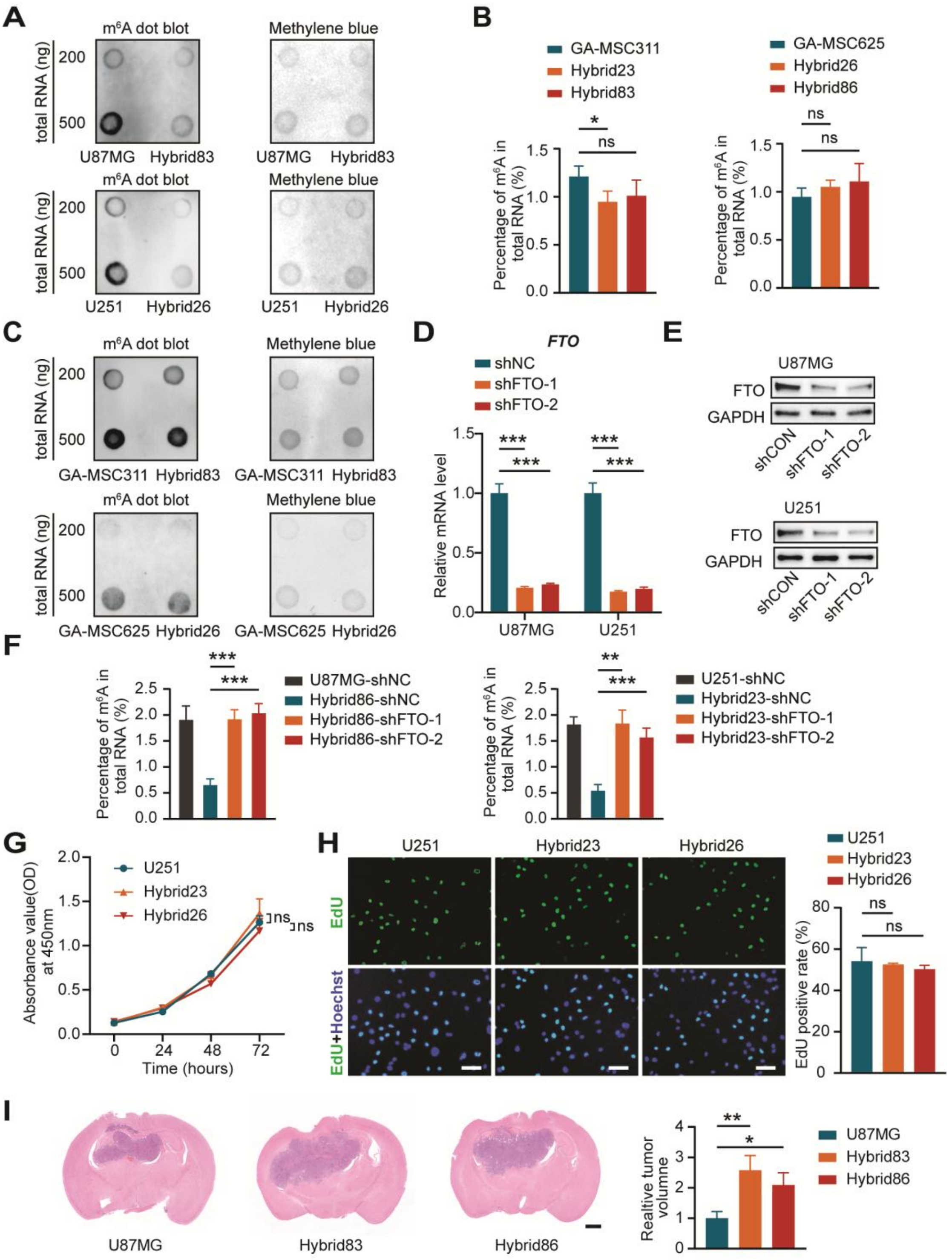

Fig. S3 The hybrids show reduced levels of m^{6}A mediated by FTO. (A) Dot blot analysis of m^{6}A levels in GBM cells and hybrids. (B) Detection of m^{6}A levels in GA-MSCs and hybrids by measuring the percentage of m^{6}A in total RNA using the m^{6}A RNA methylation quantification kit. (C) Dot blot analysis of m^{6}A levels in GA-MSCs

and hybrids (D, E) Detection of FTO expression in U87MG and U251 cells transfected with shFTO-1/shFTO-2 using qPCR (D) and western blotting (E). (F) Measurement of m^6A levels in hybrids following FTO knockdown using the m^6A RNA methylation quantification kit. (G) Assessment of proliferation in U251, Hybrid23 and Hybrid26 using the CCK-8 assay. (H) Representative images and statistical results of EdU assays demonstrating the proliferation of U251, Hybrid23 and Hybrid26. Scale bar: 100 μm. (I) Representative images of H&E staining and statistical results 3 weeks after the implantation of cells from each group. Scale bar: 1mm.

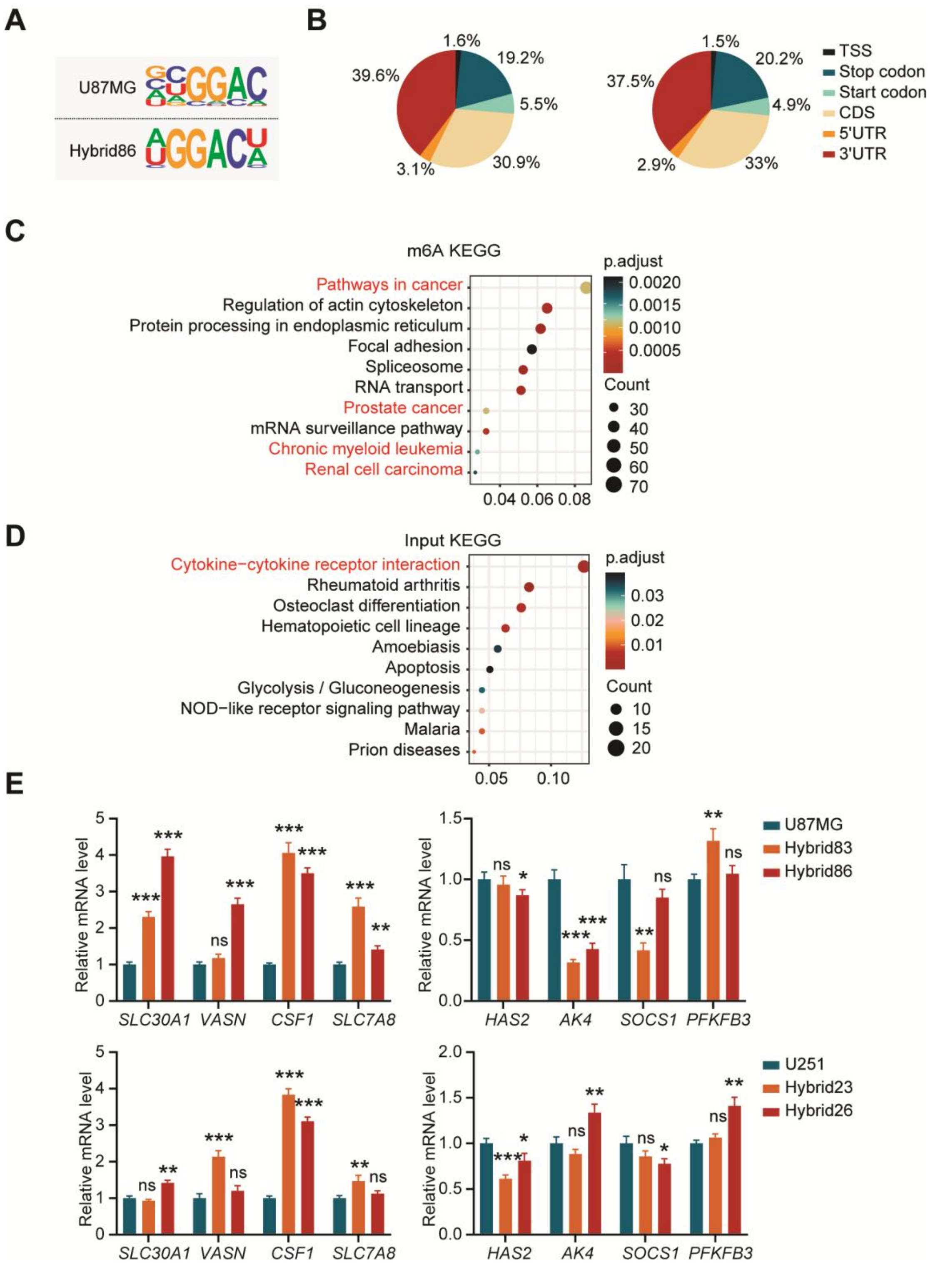

Fig. S4 Analysis of the m6A-seq data. (A) Top consensus motif of Me-RIP-Seq peaks of transcripts in U87MG and Hybrid86. (B) Proportion of m^{6}A peak distribution in the TSS, stop codon, start codon, CDS, 5′ UTR, or 3′ UTR of mRNA transcripts. (C) Bubble diagram showing the results of KEGG pathway enrichment

analysis for genes with differential m^{6}A modifications. (D) Bubble diagram showing the results of KEGG pathway enrichment analysis for differentially expressed genes. (E) qPCR analysis of SLC30A1, VASN, CSF1, SLC7A8, HAS2, AK4, SOCS1, and PFKFB3 expression in GBM cells and hybrids.

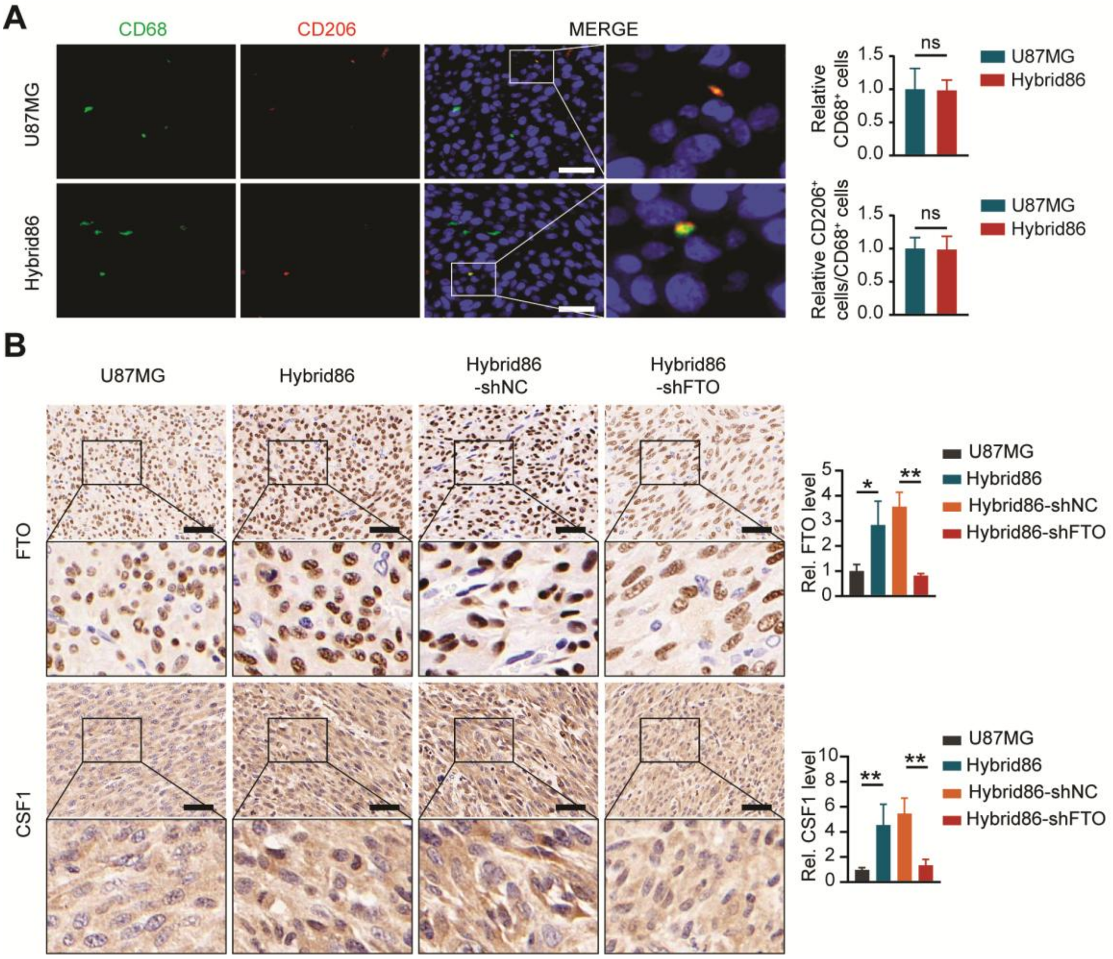

Fig. S5 Detection of macrophage markers and FTO/CSF1 levels under different conditions. (A): Representative images and statistical results of IF staining showing the distribution and proportion of CD68 and CD206 positive cells in the different groups treated with clodronate. Scale bar: 40 μm. (B): Representative images and statistical results of IHC staining for FTO and CSF1 levels in different groups. Scale bar: 20 μm.

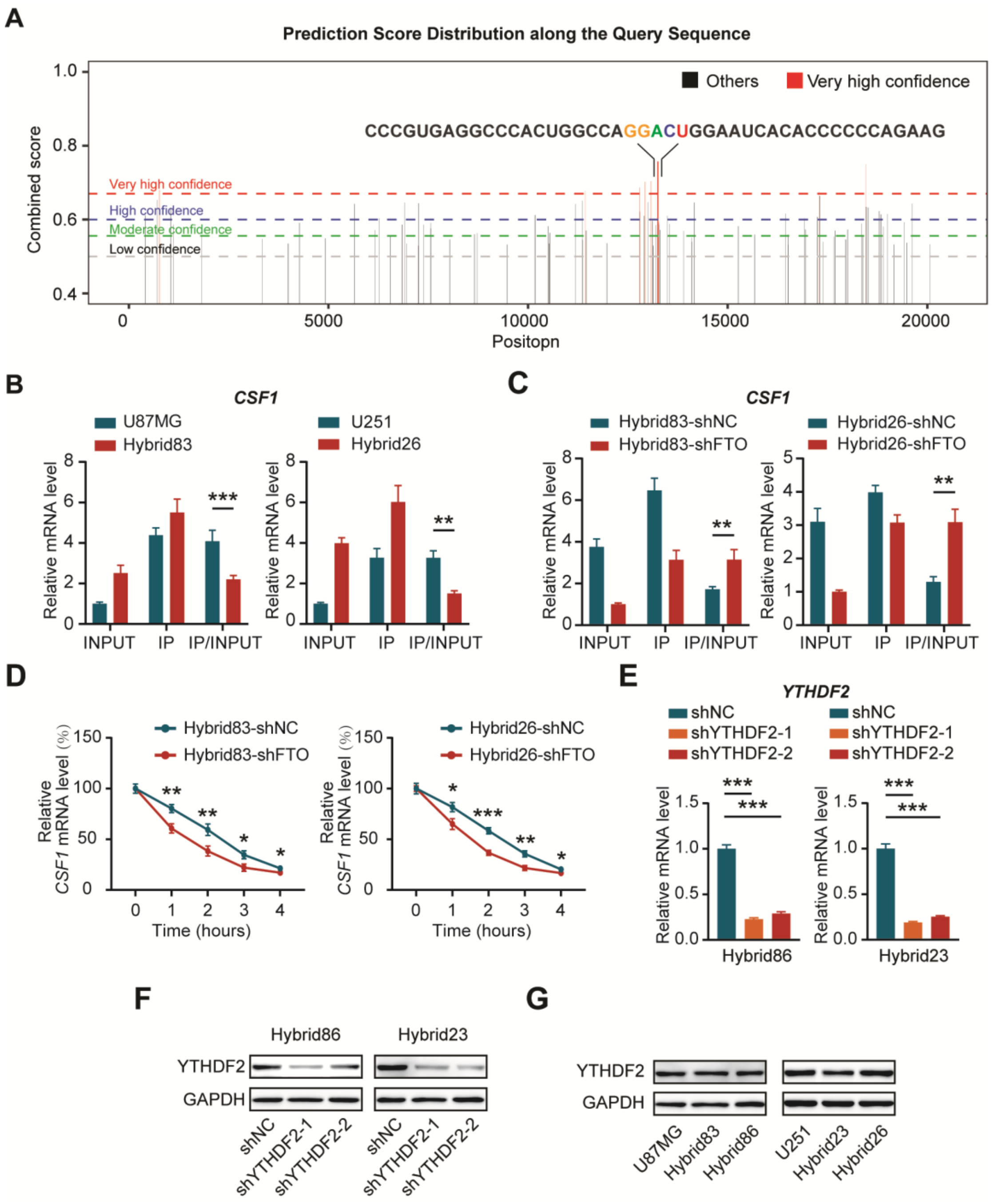

Fig. S6 FTO regulates CSF1 expression in an m^{6}A-YTHDF2-dependent manner.

(A) Distribution of predicted m^{6}A modification sites on CSF1 mRNA as identified by SRAMP. (B) Measurement of m^{6}A modification levels of CSF1 mRNA in GBM cells and hybrids using the MeRIP m^{6}A Kit. (C) Detection of m^{6}A modification levels of CSF1 mRNA in control and FTO knockdown hybrids using the MeRIP m^{6}A Kit. (D) qPCR analysis of CSF1 mRNA levels in control and FTO knockdown hybrids after

actinomycin D treatment for 0, 1, 2, 3, or 4 hours. (E, F) Verification of the knockdown efficiency of YTHDF2 in hybrids by qPCR (E) and western blotting (F). (G) Western blotting analysis of YTHDF2 expression in GBM cells and hybrids.

Additional file 2: Western blots images

Fig. 2F

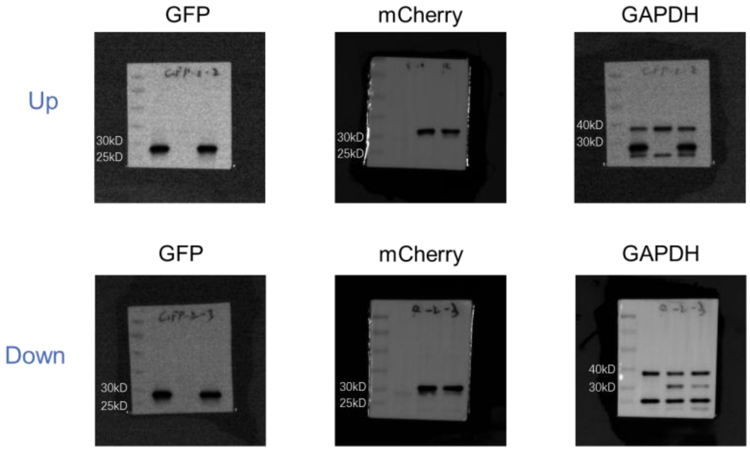

Fig. 3C

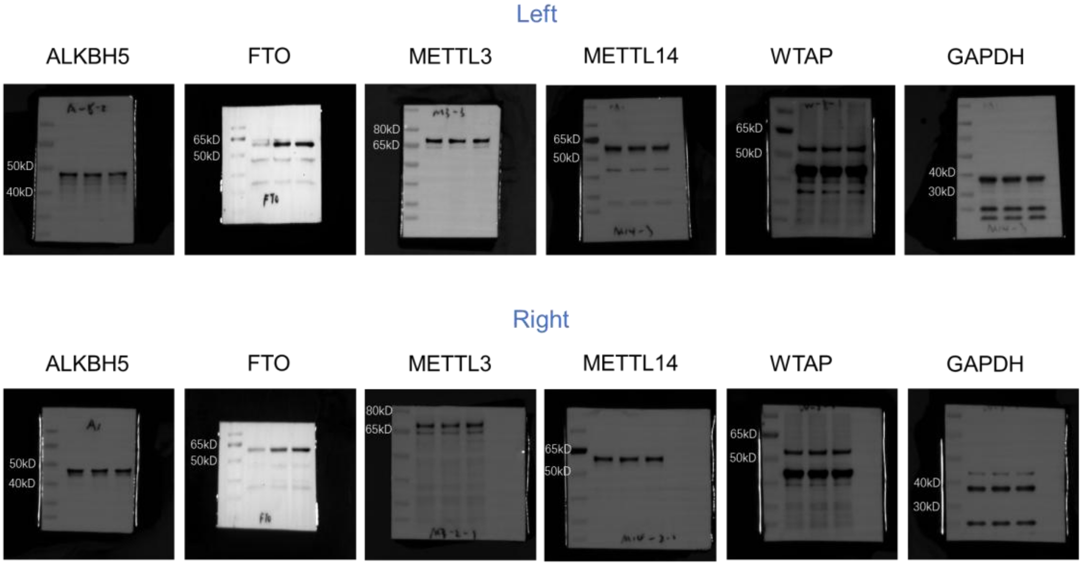

Fig. 4C

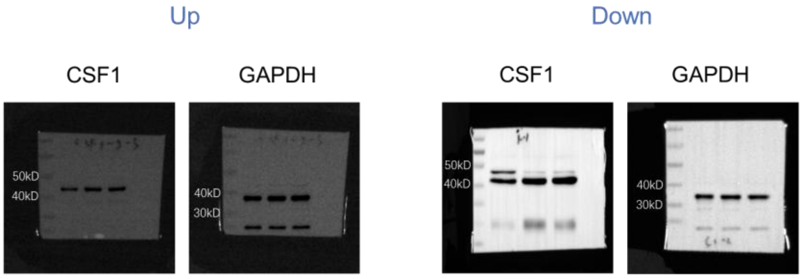

Fig. 4F

Left

CSF1

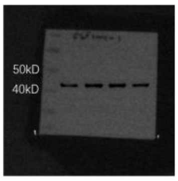

FTO

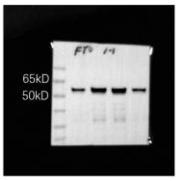

GAPDH

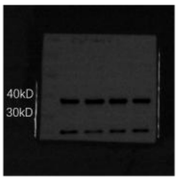

Right

CSF1

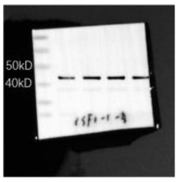

FTO

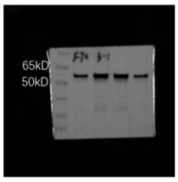

GAPDH

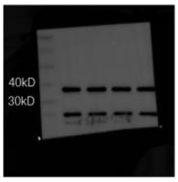

Fig. 5B

Up

CD206

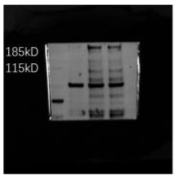

GAPDH

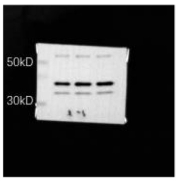

Down

CD206

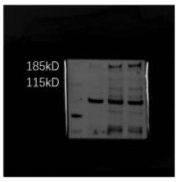

GAPDH

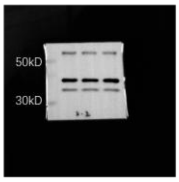

Fig. 6B

Left

CD206

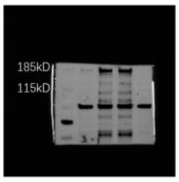

GAPDH

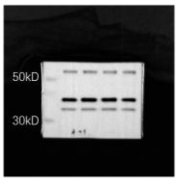

Right

CD206

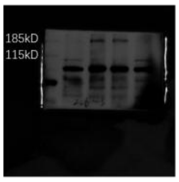

GAPDH

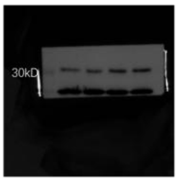

Fig. S3E

Up | Down

FTO | GAPDH | FTO | GAPDH

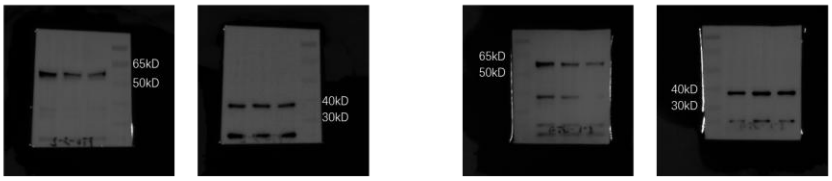

Fig. S6F

Left | Right

YTHDF2 | GAPDH | YTHDF2 | GAPDH

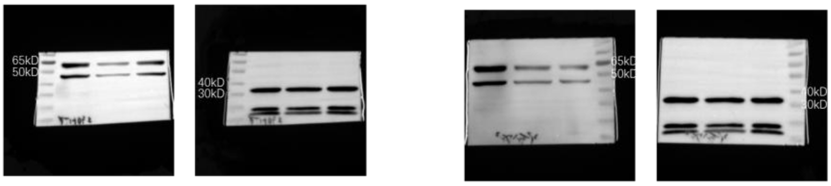

Fig. S6G

Left | Right

YTHDF2 | GAPDH | YTHDF2 | GAPDH

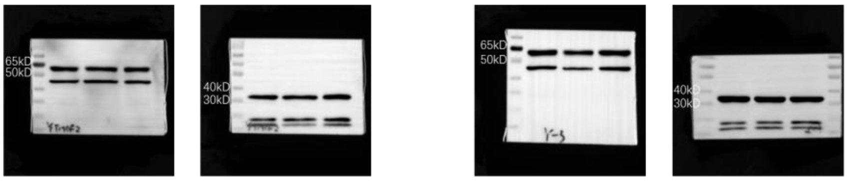

Supplement: Supplementary file 2 — Additional files [file 41419_2025_7678_MOESM2_ESM.pdf]
